# Characterizing the immune infiltrate in secondary syphilis: implications for transmission and pathology

**DOI:** 10.3389/fimmu.2025.1549206

**Published:** 2025-03-25

**Authors:** Irène Gallais Sérézal, Joseph Kirma, Mrinal K. Sarkar, Christopher Cole, Xianying Xing, Rachael Bogle, Jennifer Fox, Anthony Coon, Kelsey R. vanStraalen, Craig Dobry, Linda H. Xu, J. Michelle Kahlenberg, Paul W. Harms, Allison C. Billi, Lam C. Tsoi, Lorenzo Giacani, Johann E. Gudjonsson

**Affiliations:** ^1^ Department of Dermatology, Institut National de la Santé et de la Recherche Médicale (INSERM) 1098, Franche Comté University, Besançon University Hospital, Besançon, France; ^2^ Department of Dermatology, University of Michigan, Ann Arbor, MI, United States; ^3^ Laboratory for Experimental Immunodermatology, Department of Dermatology, Erasmus University Medical Center, Rotterdam, Netherlands; ^4^ Department of Medicine, Division of Allergy and Infectious Diseases, University of Washington, Seattle, WA, United States; ^5^ Department of Internal Medicine, Division of Rheumatology, University of Michigan, Ann Arbor, MI, United States; ^6^ Department of Pathology, University of Michigan, Ann Arbor, MI, United States; ^7^ Department of Computational Medicine and Bioinformatics, University of Michigan, Ann Arbor, MI, United States

**Keywords:** syphilis, immune evasion, keratinocytes, transcriptome (RNA-seq), host-pathogen adaptation

## Abstract

**Introduction:**

Syphilis is a complex disease with variable clinical presentation where symptomatic and potentially infectious stages alternate with periods of latency, representing a fascinating model to study immune evasion and host immune responses.

**Methods:**

Immunohistochemistry (IHC), bulk, and single-cell RNA sequencing were performed on formalin-fixed paraffin-embedded skin biopsies collected from subjects with secondary syphilis. Additionally, PBMCs from healthy individuals and either primary or *MyD88* knock-out keratinocytes were exposed to live *Treponema pallidum* cells to define initial skin responses to the bacteria.

**Results:**

Immunohistochemistry of secondary syphilis skin lesions showed a polymorphous immune infiltrate with colocalization of T cells, B cells and antigen–presenting cells. Single-cell analysis revealed distinct cellular contributions to the immune response, with prominent immune-stromal crosstalk accompanied by altered keratinocyte differentiation and decreased intraepidermal communication. Notably, prominent inflammatory signals were countered by concomitant regulatory responses, particularly in infiltrating myeloid cells. Exposure of PBMCs to live *T. pallidum* inhibited immune responses, while exposure to sonicated cells triggered *CXCL1* and *CXCL3* upregulation. Keratinocytes responded to both intact and sonicated *T. pallidum* with upregulation of type-I interferon responses that, however, were abolished in MYD88–deficient but not in STING–deficient keratinocytes.

**Discussion:**

Our data provide novel insights into the contribution of epidermal TLR sensing through MYD88 to the host response to syphilis infection, highlighting mechanisms by which *T. pallidum* evades immune responses in skin that may facilitate transmission of this pathogen through the skin.

## Graphical Abstract

S. syphilis, secondary syphilis; PBMCs, peripheral blood mononuclear cells.

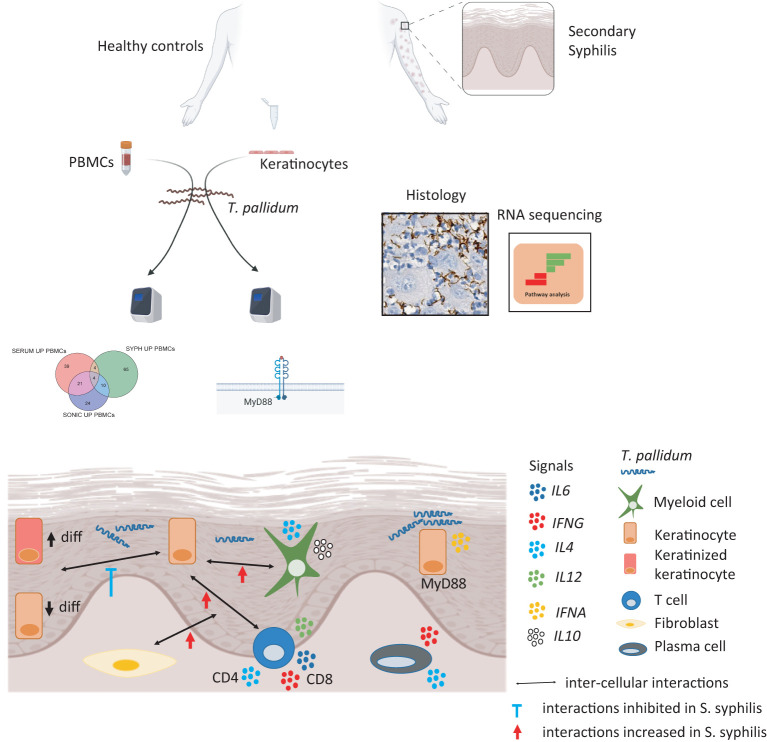

## Introduction

Syphilis remains a significant public health issue (1), particularly in low- and middle-income countries with substandard access to healthcare. Syphilis is a complex disease with protean clinical presentation, proceeding through stages that contribute to the difficulties and delays in diagnosis and treatment. The alternation of symptomatic stages and periods of latency typical of syphilis infection makes it a fascinating disease model to study immune evasion, temporal modulation, and development of immunity to the pathogen. A better understanding of the pathogenicity of the syphilis agent, *Treponema pallidum* subsp. *pallidum* (*T. pallidum* hereafter) and the associated host response are fundamental to devising novel control strategies and contribute to the development of an effective preventative vaccine (2).

The motility of the spirochete *T. pallidum* relies on flagella concealed under a low-immunogenic outer membrane layer, known to be devoid of protein antigens (3, 4). *T. pallidum* infects a new host through the skin or mucosa, causing a painless primary lesion (the chancre), followed by a maculopapular rash and a range of associated systemic symptoms during the secondary stage. Following early symptoms resolution, the infection becomes latent. If left untreated, secondary manifestations can reoccur, while tertiary syphilis may develop following a period of latency lasting up to several decades (1).

In secondary syphilis, the cutaneous immune response to *T. pallidum* exhibits a polymorphous infiltrate, including T cells, plasma cells, dendritic cells (DC), macrophages, and NK cells (5–7). The nature of the immune organization has been proposed as lymphoid-like with a transcriptomic cytotoxic Th1 signature, contrasting with the extracellular localization of *T. pallidum* (7–10). However, a thorough characterization of the cellular functions has been lacking, including the involvement and nature of the local epithelial and stromal cells in the pathogenetic processes. This may provide insights into the mechanisms by which *T. pallidum* achieves immune evasion and disease latency.

## Patients and methods

### Patients samples

Formalin-Fixed Paraffin–Embedded (FFPE) samples from diagnostic 4mm-biopsies were obtained through clinical care. Biopsies were taken as part of the diagnostic process, and none of the patients had received treatment at the time of biopsy collection. Syphilis diagnosis was based on the evaluation of clinical, serological, and histological findings, including spirochete stain, along with a positive rapid plasma regain (RPR) test. All patients tested negative for HIV. Control 6mm-skin biopsies were obtained from healthy volunteers. The University of Michigan Institutional Review Board (IRB) approved the study (HUM00087890 and HUM00174864-JEG), which was conducted in accordance with the Declaration of Helsinki Principles. Diagnosis of syphilis was confirmed by Fontana’s method, patient information is presented in **Supplementary Table 1**.

### Immunohistochemistry

FFPE skin tissue sections from biopsies of secondary syphilis patients and healthy controls were heated at 60°C for 30 minutes, de-paraffinized, and rehydrated (11). Slides were heated in pH6 or pH9 antigen retrieval buffer at 125°C for 30 minutes in a pressure cooker water bath. After treatment with 3% H_2_O_2_ (5 min) and blocking using 10% goat serum (30 min), sections were incubated overnight at 4°C with the primary antibody (**Supplementary Table 2**). Slides were then washed prior to addition of the appropriate peroxidase-labelled secondary antibody, washed again, and then developed using a diaminobenzidine substrate for 30 min.

### Exposure of keratinocytes and PBMCs to *T. pallidum*

To account for strain-to-strain variability, three *T. pallidum* strains (Chicago, SS14, Nichols) were used in this study *T. pallidum* strains were grown *in vitro* and harvested as previously described (12). Treponemal suspensions were provided at a concentration of 8.8x10 (7) cells/ml (Nichols), 1.2x10^8^ cells/ml (SS14), and 2.0x10^8^ cells/ml (Chicago) in serum-based TpCM2 growth media supplemented with 20% sterile glycerol to preserve viability. Prior to freezing, treponemal motility and integrity was assessed via dark-field microscopy, and only cell suspensions with >99% of motile treponemes were used to make frozen stocks. NTERT-2G keratinocytes knocked-out for STING (1 cell-line) or MyD88 (1 cell-line) were used as previously described (13). Each well of a 24-well plate was seeded with 10,000 cells, in KC-SFM medium (ThermoFisher #17005-042) supplemented with 30 µg/ml bovine pituitary extract, 0.2 ng/ml epidermal growth factor, and 0.3 mM calcium chloride. After reaching confluence, the cells were exposed to *T. pallidum*. For methods development, low (10,000 cells/mL), medium (100,000 cells/mL), or high (1,000,000 cells/mL) concentrations of *T. pallidum* were tested at two different time points (6 and 24 hours). Only the highest concentration and the Chicago strain was used for the main figures (see **Supplementary Figure 1**). Whole blood was obtained from three donors, and PBMCs were purified on Ficoll-Hypaque (Pharmacia Fine Chemicals, Uppsala, Sweden), and subsequently exposed to *T. pallidum* as described above in a 24 well-plate containing 400,000 cells per well.

### RNA purification and bulk sequencing

Five 20-µm sections were obtained from FFPE skin biopsies. For bulk sequencing: nine skin samples from syphilitic patients and three control samples were used. For single cell sequencing (SCC), five patient skin samples and four healthy control samples were used, were used.

RNA isolation was performed using the Qiagen RNeasy FFPE Kit (cat. no. 73504). Libraries were prepped using the QuantSeq 3’ mRNA-Seq Library Prep Kit. Libraries were then sequenced using standard procedures on the Illumina NovaSeq 6000 SP Flow Cells. Data were analyzed with the Scientific Data Analysis Platform (SciDAP; Datirium) (17): QuantSeq 3’ mRNA-Seq single-read was used to trim adapters, map reads to GRCh38.p14 (hg38), and quantify gene expression. DESeq2 (18) was used to perform differential expression analysis. Genes with a log2FC |≥1| and FDR <0.1 were considered significantly differentially expressed genes (unless otherwise stated in the manuscript). RNA from PBMCs and keratinocytes was processed using Qiagen RNeasy kit and analyzed as abovementioned.

### Single-cell RNA-sequencing

Single cell RNA sequencing was performed using 10X Genomics Flex-seq kit. Tissue was dissociated per the manufacturer’s instructions using the gentleMACS Octo Dissociator. In brief: 50um scrolls were placed into a GentleMACS C-tube (Miltenyi BioTech). The scrolls were then deparaffinized, washed and dissociated into a single cell suspension enzymatically using Liberase TH. Reads were aligned to the GRCh38 genome. CellRanger 7.0.1 was used to generate the gene x cell matrix. Matrices were loaded into Seurat v4.3.0. Ambient RNA was removed using SoupX v1.6.2 using default settings. ScDblFinder v1.12.0 was used to remove doublets using default settings. Cells containing fewer than 200 UMI and more than 25% mitochondrial reads were removed. Seurat v4.3.0 was used to normalize, scale, and reduce dimensionality of the data using Uniform Manifold Approximation and Projection (UMAP). Batch correction was performed using harmony v 1.0, using donor as batch. Clusters were annotated manually using a list of literature-based marker genes.

### Ligand–receptor interaction analysis

CellphoneDB (v2.0.0) (14) was applied for ligand-receptor analysis. The potential ligand-receptor pairs were identified using Seurat normalized counts and sub-cluster annotation as input. Pairs with p-value >0.05 were filtered out. The sub-clusters were divided into Control-specific sub-cluster and a Syphilis-specific sub-cluster. CellphoneDB was then run on the syphilis cells and on the control cells. The pairs from the sub-clusters for the same cell type were merged to find ligand-receptor pairs between the major cell types. The pairs with higher interaction scores were plotted. For the ligand-receptor analysis restricted to immunoglobulins and complement, a custom list of interactions was added to the default CellphoneDB reference panel using GRCh38 annotations.

## Results

### Transcriptomic characterization of the overall immune signals in secondary syphilis secondary skin lesions

**Figure 1:**
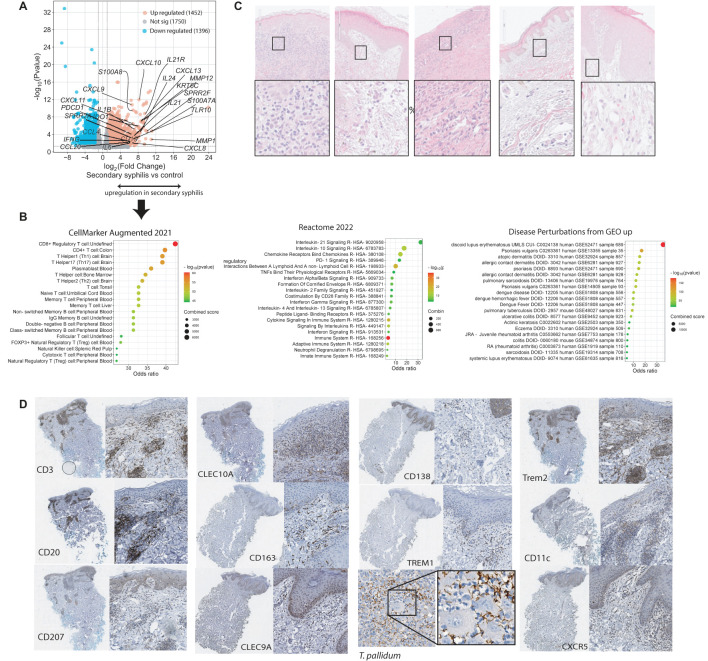
Transcriptomic characterization of the overall immune signals in secondary syphilis. Paraffin–embedded blocks were sectioned for bulk RNA sequencing n=9 and compared to healthy controls without skin diseases n=8. **(A)** volcano plot of the upregulated (red) and downregulated transcripts with fold change (FC) >2 or <-2. **(B)** Gene Set enrichment of the upregulated transcripts in **(A)**. **(C)** H&S staining of the five clinical biopsies analysed in bulk sequencing, Black squares indicate the magnified areas. **(D)** Immunohistochemistry staining of selected secondary syphilis biopsies. The complete area of the biopsy and a higher magnification insert are depicted. *T. pallidum, Treponema pallidum*. Significantly different transcripts with FDR<0.05.

Bulk RNA sequencing was performed on skin biopsies with positive staining for treponemes (not shown) from five patients with secondary syphilis. A total of 1,452 upregulated and 1,396 down-regulated genes were identified (Log2 fold-change (FC) >1, or <-1, FDR<=0.05, **Figure 1A**) when compared to healthy skin samples. *IL21*, *CXCL13* were among the top upregulated genes, both of which are involved in B-cell homing and activation (40). Increased expression of *IFNG*, *CXCL9*, *CXCL10* indicated Th1 cell activation. Evidence for matrix remodeling was reflected in the upregulation of the metalloproteinases *MMP1* and *MMP12*. Lastly, upregulation of SPPR2 family members and *S100A7* suggested an impact on epidermal differentiation. Pathway analysis (Reactome) demonstrated upregulation of IL-21, IL-10, and IFN signaling (**Figure 1B**). Cellular signals included enrichment for CD4 and CD8 T cells, which were more prominent than the transcriptomic signature for B cells (**Figure 1B**). Enriched disease-perturbation analysis showed overlap with a broad range of transcriptomic signatures of skin diseases, including discoid lupus, psoriasis, and eczema (**Figure 1B**). We validated some of these findings by immunohistochemistry, where samples exhibited a highly variable immune infiltrate (**Figure 1C**), and showed major T-cell, B-cell, and DC infiltrates in syphilis skin (**Figure 1D**), consistent with previous reports (7, 15). Prominent co-localization was observed with the T cells (CD3), B cells (CD20), and antigen-presenting cells (CD11c, TREM2, CLEC9A) being in close proximity in syphilis skin lesions (**Figure 1D**).

### Distinct cell contributions to the inflammatory environment of secondary syphilis secondary skin lesions

**Figure 2:**
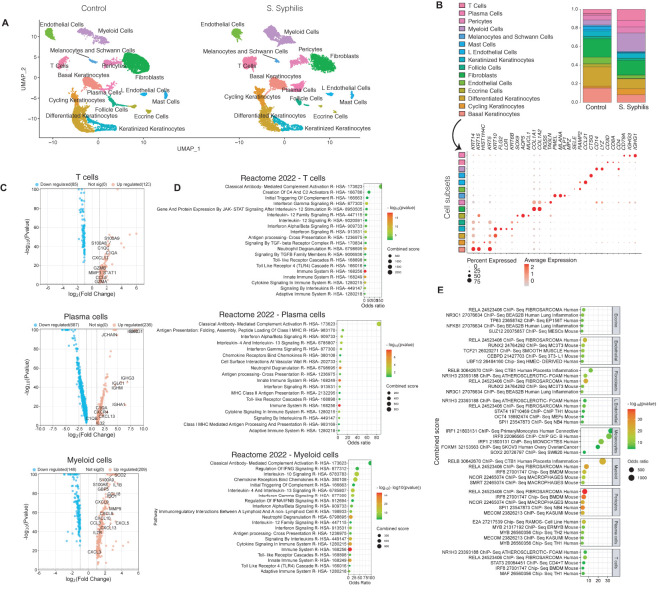
Distinct cell contributions to the inflammatory environment of secondary syphilis. **(A)** UMAP plot of the cells retrieved from secondary syphilis skin biopsies and healthy controls showing the 15 subsets identified. **(B)** Proportions of the 15 cellular subsets (up) and the markers used for the identifications (down). **(C)** Volcano plot of the upregulated (red) and downregulated transcripts with FC >2 or <-2, comparing syphilitic vs. healthy skin. **(D)** Gene Set enrichment of the upregulated transcripts. **(E)** Gene set enrichment analysis of transcription factors in nine of the cell types using ChEA 2022. S. syphilis, secondary syphilis. Significantly different transcripts with FDR<0.05. secondary syphilis (n=5) or controls (n=4).

Skin biopsies were processed for single-cell RNA sequencing to differentiate the respective cellular contributions to the inflammatory environment. Following quality control, our dataset encompassed 26,962 high-quality cells across 15 major cell types (**Figure 2A**). The mean gene count per cell was 1,697 and the mean read count per cell was 3,203. The expected increase in T, myeloid, and plasma cell proportions was confirmed (**Figure 2B**). A closer observation of gene expression enriched in T cells in secondary syphilis skin compared to T cells from control skin showed enrichment for complement activation and was also seen in plasma and myeloid cells. Notably, T and myeloid cells expressed high levels of the B/plasma cell- chemokine *CXCL13*, with plasma cells expressing high levels of genes involved in immunoglobulin expression (**Figure 2C**). Prominently enriched pathways shared between multiple immune cell types in secondary syphilis included type I and type II interferon signaling, IL-4/IL-13 signaling, and Toll-like receptor (TLR) activation (**Figure 2D**). Plasma cells were more inclined towards enrichment for type I interferon and IL-4/IL-13, while myeloid cells exhibited IL-10 signals and regulation of IFN-y. The predicted transcription factors identified included the NF-kB regulators, RELA/RELB, and the interferon-related transcription regulator IRF1, as most prominently enriched (**Figure 2E**).

### CD4^+^ and CD8^+^ T cells show distinct profiles but share common interaction signals with B cells

**Figure 3:**
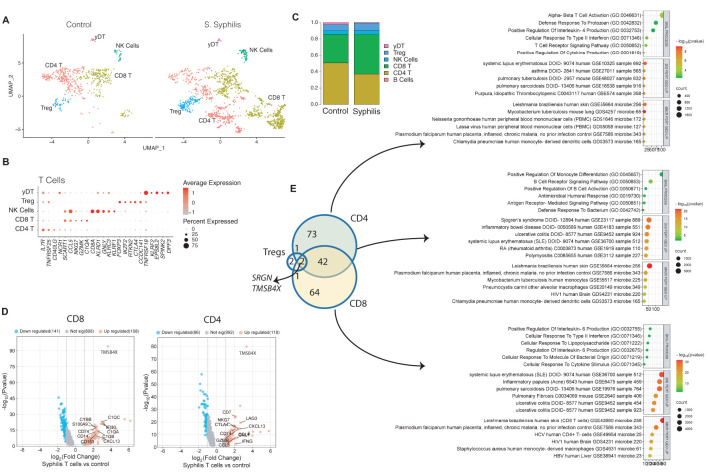
T cell profiles in syphilis skin. **(A)** UMAP plot of the 1452 T cells retrieved from secondary syphilis skin biopsies and healthy controls n=4 showing the six subsets identified. **(B, C)** Markers used for the subsets and respective cellular proportions. **(D)** Volcano plots of up (red) and down (blue)-regulated genes (DEGs). Grey dots if not statistically significant. **(E)** Venn diagram of the common upregulated DEGs between CD4, CD8, and Tregs cells, and corresponding pathway analysis using biological process, disease perturbation GEO up, and microbe perturbation GEO up, in EnrichR platform. Tregs, regulatory T cells; yDT, gamma delta T cells; NK, natural killer cells.

To differentiate T cell subsets, subclustering was performed, demonstrating distinct populations of CD4^+^, CD8^+^ T cells, regulatory T cells, and gamma delta T cells (**Figure 3A**), defined by their marker gene expression (**Figure 3B**). CD8^+^ T cells were the most enriched subset, whereas there was a decreased frequency of CD4+ T cells, with other T cell subsets showing lesser change (**Figure 3C**). To assess for changes in the biological functions of T cells in syphilis skin, we compared their differentially-expressed genes DEGs (FDR<0.05) to healthy control T cells on a per-cell type basis. Examples of DEGs in CD8 and CD4 T cells can be seen in **Figure 3D**. A total of 184 DEGs were identified, with 42 shared between CD4^+^ and CD8^+^ T cells (**Figure 3E**), with the core response of CD4 and CD8 T cells involving B cell interactions, upregulation of the B cell chemokine *CXCL13*, and Th1/Th17 disease signatures (**Figure 3E**). CD4^+^ T cells showed, in addition, a Th2 skewing with enrichment for IL-4 responses and asthma disease signature, along with upregulation of regulatory markers, including *CTLA4* and *LAG3*. CD8^+^ T cells, however, displayed enriched IL-6 and IFN-γ signaling.

### Myeloid cells in syphilis secondary skin lesions express immunomodulatory cytokines, including IL10 and TGFB

**Figure 4:**
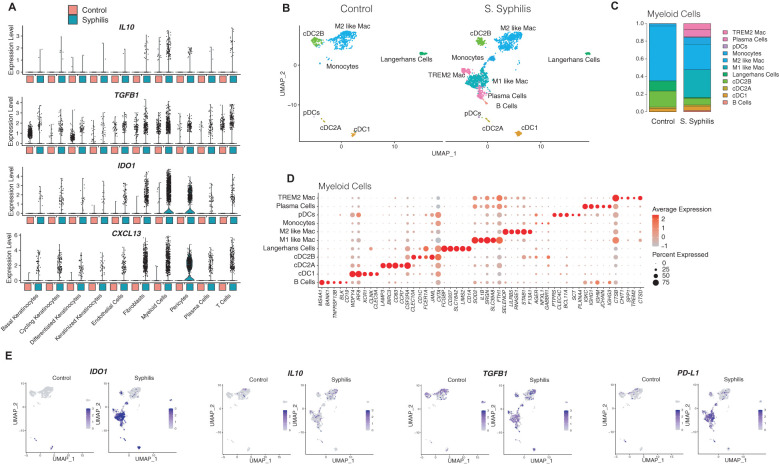
Myeloid cell function in secondary syphilis inflammatory responses. **(B)** UMAP plot restricted to 2,278 myeloid cells, with **(C)** comparison of the cell proportion between healthy skin and secondary syphilis and **(D)** the markers used to classify the myeloid cell markers. **(A)** violin plots of transcripts involved in regulatory immune responses in the major cell clusters. **(E)** UMAP plots of single markers expression in myeloid cells relevant to regulatory immune responses.

Myeloid cells in samples from syphilis patients showed prominent immunomodulatory/immunosuppressive cytokine expression, including *IL10*, *TGFB1*, *IDO1*, and the B/plasma cell chemokine *CXCL13* (**Figure 4A**). Subclustering of myeloid cells (2,667 myeloid cells from syphilis lesions, and 740 from healthy skin) showed several subsets that were restricted to syphilis samples and absent in healthy skin (**Figure 4B-D**), including TREM2 macrophages, plasmacytoid dendritic cells (pDCs), and conventional dendritic cell type 1 and types 2A and 2B (**Figure 4C**). Notably, there was a prominent decrease of Langerhans cells in syphilis skin (**Figure 4C**). B and Plasma cells clustered with the myeloid cells and increased in syphilis skin (**Figure 4C**). To assess which of the myeloid cell populations contribute to *IL10*, *IDO1*, *TGFB1*, and *PD-L1* expression, we used feature plots to determine their expression, with monocytes, M1-like macrophages, TREM2 macrophages, cDC2B being the main contributors (**Figure 4E**).

### Immune crosstalk in syphilis lesions is characterized by diminished intercellular communications in the epidermis and altered differentiation

**Figure 5:**
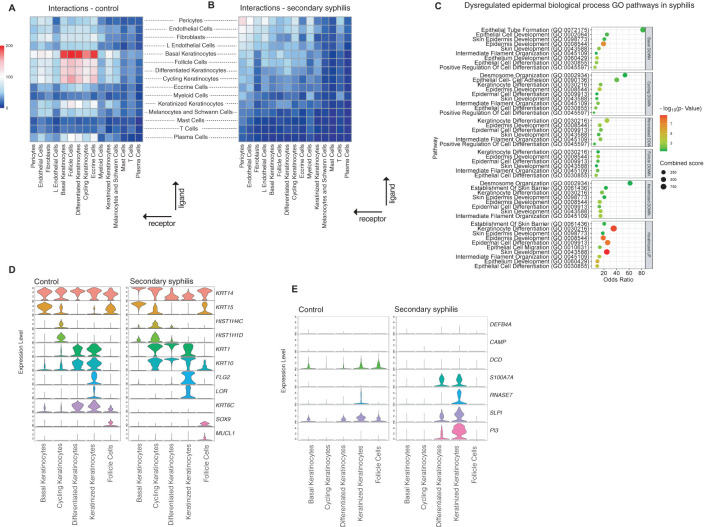
Shifts in cell-cell interactions in syphilis compared to healthy skin. **(A, B)** Heatmap of the number of ligand/receptor pairs with a higher score in secondary syphilis compared to normal skin among all the cell types identified. The ligands were expressed by the cell types in the row, and the receptors were expressed by the cell types in the column. The color scale represents the number of ligand/receptor pairs. **(C)** Gene-enrichment analysis using GO BP focused on downregulated transcripts in all keratinocyte cell subsets and upregulated transcripts in keratinized keratinocytes. **(D)** Violin plots of transcripts markers of keratinocyte differentiation and **(E)** of transcripts from genes coding for antimicrobial peptides.

To picture the intercellular interactions in secondary syphilis, ligand-receptor analysis was assessed using CellPhonedB (14). Compared to healthy skin, where most interactions were confined between keratinocyte subsets, syphilis lesions showed fewer inter-keratinocyte interactions, with no apparent immune-epithelial crosstalk (**Figure 5A, B**). Pathway analysis of the different subtypes of keratinocytes showed that differentiation was altered in all subtypes of keratinocytes concerning desmosome function and other adhesion-related mechanisms (**Figure 5C**). Distribution of differentiation markers showed a more robust expression of *KRT10* and *KRT1* in keratinized, cycling, and differentiated keratinocytes in syphilis lesions compared to healthy skin (**Figure 5D**), with the expression of the major antimicrobial peptides shifted (**Figure 5E**). The expression of *KRT14*, was redistributed: higher expression in basal keratinocytes in syphilis compared to healthy skin but lower in differentiated and keratinized keratinocytes. Surprisingly, expression of *KRT6C*, a stress-related keratin, was markedly diminished in syphilis samples compared to healthy skin (**Figure 5D**), confirming the significant impact of the infection on all keratinocyte subtypes.

### Fibroblasts exhibit a pro-inflammatory state and shift towards immunoglobulin/complement activation in syphilis secondary skin lesions

**Figure 6:**
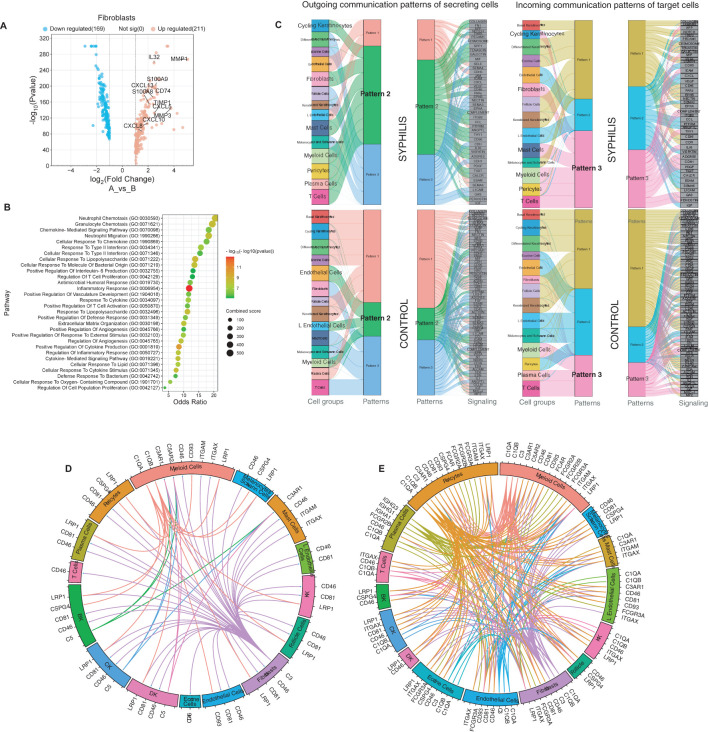
Fibroblasts and Immune-stromal interactions in secondary syphilis. **(A)** Volcano plot of the upregulated (red) and downregulated transcripts in fibroblasts, with fold change >2 or <-2, comparing fibroblasts in samples from syphilis patients and healthy controls. Significantly different transcripts with p<0.05. **(B)** Gene-enrichment analysis using the GO biological process (BP), focused on upregulated transcripts in fibroblasts. **(C)** Comparison of each cell subset’s incoming and outcoming communication patterns in samples from secondary syphilis and healthy skin. **(D, E)** Circos plot of ligand–receptor pairs restricted to complement and immunoglobulins, in healthy (left) and secondary syphilis (right).

Fibroblasts exhibited altered gene expression profile in syphilis skin, including increased expression of various chemokines, including the neutrophil chemokine *CXCL8*, Th1 chemokine *CXCL10*, and *CXCL13* (**Figure 6A**), reflecting in enriched inflammatory pathways such as neutrophil chemotaxis, response to type II IFNs, and defense response to a bacterium (**Figure 6B**). Further analysis of the outgoing communication pattern showed that myeloid and endothelial cells were part of a broad interaction web, including cycling keratinocytes and other immune cell subsets (**Figure 6C**). The incoming communication patterns showed that myeloid and T cells shared similar interactions with fibroblasts and cycling keratinocytes that is absent in healthy skin. Next, given the hallmark B and plasma cell infiltration in syphilis lesions, we focused on complement and immunoglobulin–related interactions to determine if those could be a driver of the immune-stromal crosstalk in the host response to *T. pallidum*. Thus, a marked increase was observed in stromal-immune interactions in syphilis skin, with fibroblasts, endothelial cells, and pericytes being a significant source of complement components as well as expressing receptors for plasma cell-derived immunoglobulins (**Figure 6D, E**). This suggests that a major feature of immune-stromal crosstalk in syphilis secondary skin lesions is centered around immunoglobulin/complement activation.

### Intact *T. pallidum* inhibits innate immune responses in PBMCs while sonicated bacteria trigger an inflammatory response

**Figure 7:**
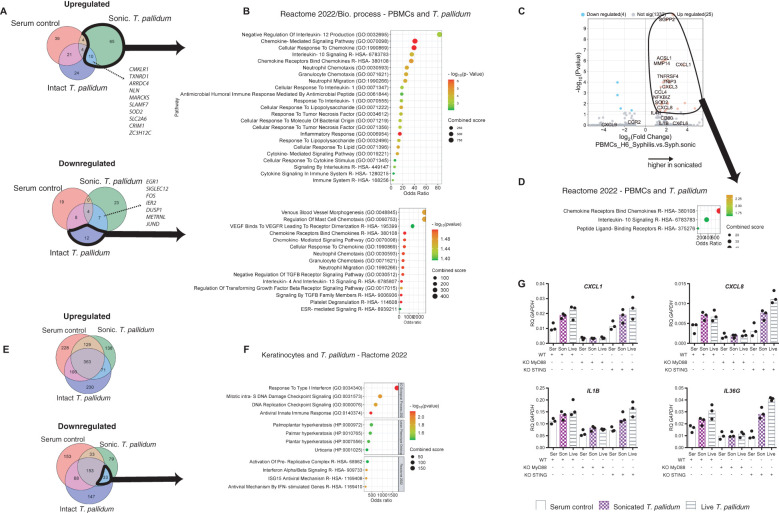
Inflammatory responses in *In vitro* in human PBMCs to *Treponema pallidum* and role of MyD88 in keratinocytes. **(A)** Comparison of the gene expression in PBMCs at baseline and after 6 hours for each of the three conditions. Venn diagram of the upregulated transcripts, showing overlapping significantly upregulated (up) or downregulated (down) transcripts. **(B)** Gene set enrichment analysis of the identified transcripts circled in black in **(A)**, using Reactome 2022 gene set. **(C)** Volcano plots of dysregulated transcripts after 6 hours of sonicated *T. pallidum* compared to serum exposure, on the left, and of dysregulated transcripts after 6 hours of sonicated *T. pallidum* compared to live *T. pallidum* exposure (middle) and the corresponding pathway analysis using Reactome 2022. **(E)** Comparison of the gene expression in keratinocytes at baseline and after 6 hours, for each of the three stimulations. Venn diagram of the upregulated transcripts, showing overlapping significantly upregulated (down) or downregulated (up) transcripts. **(F)** Gene set enrichment analysis of the identified transcripts circled in black in **(E)**, using Reactome 2022 gene set. **(G)** MyD88^-/-^ or STING^-/-^ keratinocytes and wild-type keratinocytes, were exposed for 6 hours to *T. pallidum* cells, either live or sonicated, or the equivalent volume of rabbit serum. RT-qPCR was performed after the purification of mRNA. Kc, keratinocytes, son/sonic, sonicated, indicates exposure to sonicated *T. pallidum.* Syph/syphilis, indicates intact *T. pallidum* exposure, RQ, relative quantification, WT, wild type, KO, knock-out, ser, rabbit serum control. Data representative of n=3.

Secondary syphilis is a systemic phase of the disease in which treponemes disseminate from the primary chancre to virtually all bodily organs. How *T. pallidum* escapes the innate and adaptive immune responses is only partially understood. Therefore, we exposed human PBMCs to live or sonicated *T. pallidum* and collected exposed cells after 6 hours for bulk RNA analysis (**Figure 7A**). Transcripts that were dysregulated (FDR<0.05) were used for enrichment analyses. Transcripts upregulated ((Log2 FC>=1) after 6 hours of exposure to sonicated *T. pallidum vs*. baseline (n=83) belonged to chemokine and neutrophils–related pathways, with IL-10 and negative regulation of IL-12 among the top-upregulated signatures (**Figure 7B**). Notably, only 24 transcripts were selectively upregulated in PBMCs exposed to live intact *T. pallidum*, with Neutrophil Degranulation (p=0.0009) and Innate Immune System (p=0.02975) significantly enriched. Although intact *T. pallidum* did not trigger strong immune responses in PBMCs downregulated transcripts (Log2 FC<-1) encompassed neutrophil–related pathways and chemotaxis, indicating that intact *T. pallidum* bacteria selectively inhibit immune responses within 6 hours of contact. Comparing the 6 hours–exposed samples with one another, the sonicated bacteria elicited a strong upregulation of *CXCL8*, *CXCL1*, *CXCL3*, and *CCL4* (**Figure 7C, D**).

### Human keratinocytes respond to *T. pallidum* with activation of interferon responses dependent on MyD88

Skin microabrasions can be an entry site for syphilis and is one of the sites of manifestation of secondary syphilis, where *T. pallidum* can frequently be found in the space between keratinocytes. To provide a cleaner view of the skin-specific reaction to the earliest events upon skin penetration, we exposed keratinocytes to *T. pallidum*, either live or sonicated. The number of downregulated transcripts (Log2 FC<-1, FDR<0.05) after 6 hours of exposure surpassed the upregulated ones (Log2 FC>1, FDR<0.05) (**Figure 7E**) with 421 upregulated transcripts and 764 downregulated. In the cells exposed to the sonicated bacteria, 298 upregulated transcripts were identified and 701 were downregulated. No strong proinflammatory signals were generated, and pathway analysis of upregulated transcripts identified type I interferon as the main common pattern of response between intact and sonicated bacteria (**Figure 7F**). When reproducing the experiment using keratinocytes knocked out for *MyD88*, a downstream effector of IL-1/TLR signaling, the inflammatory responses were absent, indicating a TLR–dependent mechanism of detection of treponemal cells in keratinocytes (**Figure 7G**), as previously published (16–18).

## Discussion

Our data provide some insights into the mechanisms involved and the sequence of events that drive the unique histological features of secondary syphilis, including the rich B- and plasma cell infiltrates. It sheds light on the ability of *T. pallidum* to invade and multiply in the skin and mucosa that is crucial to its contagiousness and also generates hypothesis regarding how the bacteria evades immune response and establishes latency.

A striking finding of this work was the response of epidermal keratinocytes to *T. pallidum*. Overall, the keratinocyte response to both live and sonicated *T. pallidum* were more modest than those of PBMCs. Our data confirm the dependence of keratinocyte immune responses to *T. pallidum* on *MyD88*. MyD88 is downstream of both IL-1/IL-36 and TLR responses (19, 20). Our findings align with others on the role of MyD88 in response to *T. pallidum*, particularly flagellin, in both keratinocytes (21) and PMBCs (22), and further supports the role of TLR signaling in response to syphilis infection (23, 24), which likely involves TLR2 and TLR5 responses, as shown by others (22, 24, 25). Notably, one of the most marked features observed in the single-cell sequencing data was the effect of syphilis infection on desmosome organization and epithelial cell-to-cell adhesion. Desmosomes play a crucial role in providing intercellular junctions that provide adhesions between cells to provide mechanical strength to the epidermis and skin barrier functionality (26, 27). *T. pallidum* can be identified in both the epidermis and dermis of secondary syphilis lesions (28), and these lesions are believed to be contagious by direct contact. Our data suggest that the spread of *T. pallidum* may be facilitated by these changes in adhesion structures in the epidermis, and this may be one of the mechanisms by which the bacterium promotes dissemination, although the exact mechanisms by which *T. pallidum* induces these changes in differentiated epidermis require further study.

Another important finding from our study is the characterization of the broad and mixed inflammatory responses we observe in secondary skin lesions. Secondary syphilis has been termed the “great masquerader” because its skin lesions show such diverse clinical and histological morphologies mimicking a broad range of inflammatory skin diseases (28). Our data confirm the broad immune activation in skin with both enriched anti-inflammatory mechanisms, involving IL-10, and pro-inflammatory stimuli mediated by TNF and TLR responses, type I and type II IFN responses, enriched Th2 and Th17 responses, along with neutrophil activation, B-cell chemotaxis, plasma cell activation, and enriched IgG/complement responses. Notably, even in light of T-cell phenotype, we see broad ranges overlapping with as diverse transcriptomic disease patterns as systemic lupus erythematosus (SLE), asthma, and pulmonary tuberculosis in CD4+ T cells, and acne, SLE, and sarcoidosis in CD8+ T cells from syphilis skin. Thus, it can be speculated that different balances between the effector immune cells in syphilis skin can lead to dramatically different clinical manifestations despite the same underlying disease mechanisms.

As described before, the inflammatory infiltrate in syphilis is very localized, involving complex lymphoid-like collections that often surround the superficial blood vessels in the skin. The presence of tertiary lymphoid structures (TLS) in secondary syphilis has been suggested (8), and may contribute to the progression of immune responses in skin. The increased *CXCL13* mRNA expression we detected aligned with increased infiltration of CXCR5+ cells, but CXCR5 can be seen on B cells, Tfh cells (29, 30), and follicular dendritic cells (31, 32). Our data is also consistent with an inflammatory network characterized by the production of immunoglobulins and complement components. While plasma cells are the source of both IgG and IgA antibodies, other cell types, particularly stromal cell types, contributed both as potential targets of the IgA antibodies through Fc receptor expression or expression of various complement components or receptors. Circulating immune complexes that contain treponemal antigen together with antibody and complement have been shown to be active in secondary syphilis (33–36).

Moreover, our data may provide some indications by which *T. pallidum* may evade clearance. Previous work has suggested that *T. pallidum* displays several strategies for immune evasion. This includes their slow growth (36-44 hour generation time), antigenic variation, and the paucity of surface-exposed antigens on the pathogen outer membrane, which shields highly immunogenic/immunodominant sub-surface antigens such as lipoproteins and flagellar components, which delays the initial host response (5). The presence of this outer membrane explains our findings of stronger immune responses triggered by the sonicated bacteria than intact ones. However, “active inhibition” of the immune system could be witnessed after exposing PBMCs to live *T. pallidum*, which could not be explain by a lack of detection of surface antigens. Indeed, other mechanisms are suggested in the literature and include interference with immunosuppressive or immunomodulatory mechanisms such as IL-10 or TGF-B. *T. pallidum*–specific antigens have been shown to promote IL-10 production and TGF-B in monocytes (37), which is consistent with our data showing that expression of genes involved in IL-10 signaling is significantly altered in secondary skin lesions. Other mechanisms that could contribute to this and suggested in our data include the altered keratinocyte differentiation and relative paucity of antimicrobial responses, which may limit the local bacterial clearance (38). Also, the differences between sonicated and intact bacteria could depend on the inactivation of heat-sensitive proteins, as described in *Treponema denticola* (39). Lastly, myeloid cells may also contribute through mechanisms involving *IDO*, *TGFB1*, or *PD-L1*, which were all dramatically upregulated in syphilis, with each one providing a potential mechanism contributing to immune evasion.

Several limitations should be acknowledged. First, the number of samples that were analyzed with single-cell RNA sequencing is low, and the cellular composition of the paraffin–embedded tissue might not reflect all cell types actually present in the skin at the time of the biopsy collection. However, quality control reached satisfying levels and many different cell types were identified. Also, the body sites of the skin biopsies for the single-cell sequencing were more heterogenous in patients compared to the healthy controls that were taken from the hip. Additionally, patients were all male whereas the majority of controls were female. While this could have contributed to some of the differences identified in the data, we think it is unlikely to have a major impact on our findings. Finally, monolayer cultures of keratinocytes are not as physiological as actual human skin tissue, but allowed for a first characterization of the keratinocytes responses to *T. pallidum* in both WT and KO keratinocytes.

In conclusion, our data highlight the broad and mixed inflammatory patterns in syphilis secondary skin lesions and emphasize how this disease is an excellent model for studying host-pathogen interactions. Our data provide insights into some of the mechanisms by which syphilis may evade immune responses as well as those that may facilitate transmission through the skin.

## Data Availability

The original contributions presented in the study are publicly available. This data can be found here: NCBI - The single-cell data is under GSE292160, and the bulk data is under GSE261255.

## Supplementary material

Supplementary Figure 1 time-course of cytokine upregulation and effect of strains and concentration of T. pallidum on human PBMCs and keratinocytes. The upper panel shows the strain effect of T. pallidum upon exposure of WT Keratinocytes to the bacteria. The lower panel show the upregulation of IL1B at 6 hours versus 24 hours, and with three different concentrations (low, medium, high).

Supplementary Table 1 patients and healthy controls.

Supplementary Table 2 antibodies used for immunohistochemistry.
