# Supplementary material for: Characterizing the immune infiltrate in secondary syphilis: implications for transmission and pathology
Source: Front Immunol. 2025 Mar 25;16:1549206. doi: 10.3389/fimmu.2025.1549206 (PMC11975926; doi:10.3389/fimmu.2025.1549206)
Supplement: Supplementary Figure 1 — time-course of cytokine upregulation and effect of strains and concentration of T. pallidum on human PBMCs and keratinocytes. The upper panel shows the strain effect of T. pallidum upon exposure of WT Keratinocytes to the bacteria. The lower panel show the upregulation of IL1B at 6 hours versus 24 hours, and with three different concentrations (low, medium, high). [file DataSheet1.docx]

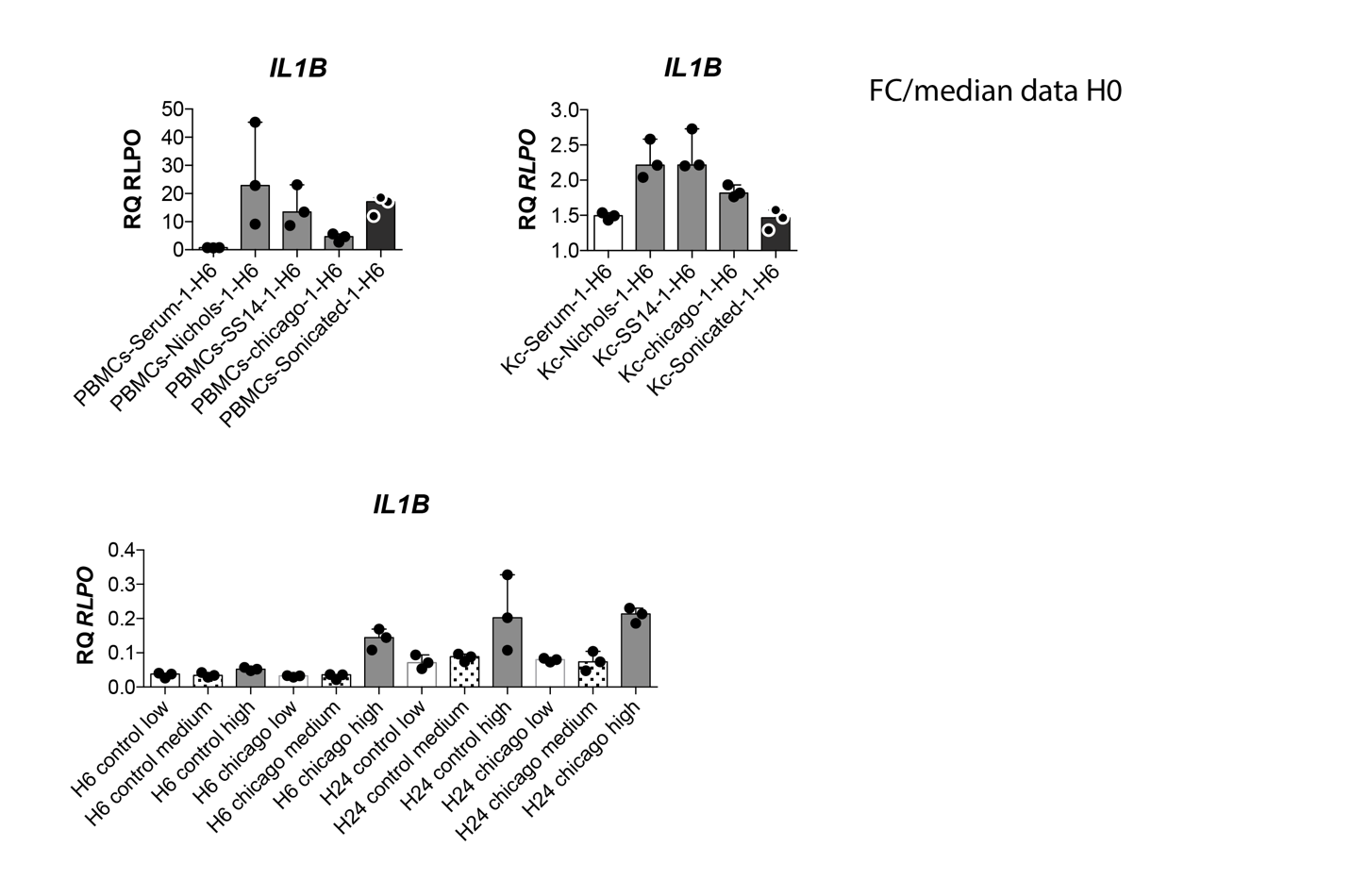


*Supplementary Figure 1: time-course of cytokine upregulation and effect of strains and concentration of T. pallidum on human PBMCs and keratinocytes*

The upper panel shows the strain effect of *T. pallidum* upon exposure of WT Keratinocytes to the bacteria. The lower panel show the upregulation of IL1B at 6 hours versus 24 hours, and with three different concentrations (low, medium, high).

| \| **Age** \| **Disease** \| **Sex** \| **Biopsy site** \| **bulkRNA-seq** \| **scRNA-seq** \| \| --- \| --- \| --- \| --- \| --- \| --- \| \| 70 \| S. Syphilis \| Male \| Left lateral anal canal \| X \| X \| \| 57 \| S. Syphilis \| Male \| Back \| X \| X \| \| 61 \| S. Syphilis \| Male \| Right palm \| X \|  \| \| 23 \| S. Syphilis \| Male \| Penile shaft \| X \|  \| \| 35 \| S. Syphilis \| Male \| Mucosa, right buccal \| X \|  \| \| 66 \| S. Syphilis \| Male \| Back \| X \| X \| \| 38 \| S. Syphilis \| Male \| Chest \| X \|  \| \| 14 \| S. Syphilis \| Male \| Left flank \| X \|  \| \| 51 \| S. Syphilis \| Male \| Skin of perianal area \| X \| X \| \| 69 \| Controls \| Female \| Hip \|  \| X \| \| 65 \| Controls \| Female \| Left Hip \|  \| X \| \| 24 \| Controls \| Male \| Left Hip \|  \| X \| \| N/A \| Controls \| Male \| Hip \|  \| X \| \| N/A \| Controls \| Male \| Hip \| X \|  \| \| N/A \| Controls \| Female \| Hip \| X \|  \| \| N/A \| Controls \| Female \| Hip \| X \|  \| \| N/A \| Controls \| Male \| Hip \| X \|  \| \| N/A \| Controls \| Female \| Hip \| X \|  \| \| 49 \| Controls \| Female \| Hip \| X \|  \| \| 47 \| Controls \| Female \| Hip \| X \|  \| \| 58 \| Controls \| Female \| Hip \| X \|  \| |
| --- | --- | --- | --- | --- | --- | --- | --- | --- | --- | --- | --- | --- | --- | --- | --- | --- | --- | --- | --- | --- | --- | --- | --- | --- | --- | --- | --- | --- | --- | --- | --- | --- | --- | --- | --- | --- | --- | --- | --- | --- | --- | --- | --- | --- | --- | --- | --- | --- | --- | --- | --- | --- | --- | --- | --- | --- | --- | --- | --- | --- | --- | --- | --- | --- | --- | --- | --- | --- | --- | --- | --- | --- | --- | --- | --- | --- | --- | --- | --- | --- | --- | --- | --- | --- | --- | --- | --- | --- | --- | --- | --- | --- | --- | --- | --- | --- | --- | --- | --- | --- | --- | --- | --- | --- | --- | --- | --- | --- | --- | --- | --- | --- | --- | --- | --- | --- | --- | --- | --- | --- | --- | --- | --- | --- | --- | --- | --- | --- | --- | --- | --- | --- |
| *Supplementary Table 1: patients and healthy controls* |

| CD163 | Thermofisher  scientific | | | MA5-11458 |  |  |  |  |  |  |  |  |
| --- | --- | --- | --- | --- | --- | --- | --- | --- | --- | --- | --- | --- |
| CLEC9A | Thermofisher  scientific | | | 55451-I-AP |  |  |  |  |  |  |  |  |
| CLEC10A | Thermofisher  scientific | | | TA810180 |  |  |  |  |  |  |  |  |
| CD207 | Thermofisher  scientific | | | PA5-82422 |  |  |  |  |  |  |  |  |
| LAMP3 | Thermofisher  scientific | | | PA5-84069 |  |  |  |  |  |  |  |  |
| CD3 | ORIGENE | | | UM500048 |  |  |  |  |  |  |  |  |
| CD20 | Abcam | | | AB9475 |  |  |  |  |  |  |  |  |
| CD138 | LIFESPAN BIOSCIENCE | | | LS-B9360 |  |  |  |  |  |  |  |  |
| TREM1 | Abcam | | | Ab225861 |  |  |  |  |  |  |  |  |
| TREM2 | Novus bio | | | NBP1-07101 |  |  |  |  |  |  |  |  |
| CD11c | abcam | | | Ab216028 |  |  |  |  |  |  |  |  |
| CXCR5 | Lifespan biosciences | | | LS-B2827 |  |  |  |  |  |  |  |  |
|  |  |  |  |  | |  |  |  |  |  |  |  |

*Supplementary Table 2: antibodies used for immunohistochemistry*
